# Development of Potentiometric Phenol Sensors by Nata de Coco Membrane on Screen-Printed Carbon Electrode

**DOI:** 10.1155/2019/4608135

**Published:** 2019-08-19

**Authors:** Ani Mulyasuryani, Afifah Muhimmatul Mustaghfiroh

**Affiliations:** Analytical Chemistry Laboratory, Department of Chemistry, Faculty of Mathematics and Natural Sciences, University of Brawijaya, Malang, Indonesia

## Abstract

Nata de coco, a bacterial cellulose as a result of coconut water fermentation, is a conductive polymer with a electrical conductivity of 553 *μ*S/cm and has high mechanical stability. In this study, nata de coco was used as a supporting membrane for the development of phenol sensors in potentiometry. Nata de coco membrane containing phenol is coated on the surface of the printed carbon electrode (screen-printed carbon electrode). The cross-sectional area of the carbon electrode coated with the membrane is 1.5 × 3 mm^2^, while the reference electrode is Ag/AgCl. The thickness of the electrode membrane affects the Nernstian factor. The optimum Nernstian factor is produced by 100 *μ*m membrane thickness containing 117.5 *μ*g of phenol. Measurement of phenol solution was carried out at pH 11, in the concentration range of 10^−8^ to 10^−2^ mol/L, resulting in a Nernstian factor of 41.8 ± 1.3 mV/decade. The Nernstian factor increased to 55.7 ± 0.4 mV/decade if the membrane of the electrode contained 0.1% Fe_3_O_4_ nanoparticles. This sensor has been applied in the real sample of river water, resulting in good accuracy and precision.

## 1. Introduction

Phenol is one of the organic substances produced from industrial plants such as petroleum. The high phenol concentration in the water system can cause death in certain groups of organisms [1]. The maximum limit of phenol concentration in the waters that is permitted based on the Regulation of the Minister of Environment of the Republic of Indonesia number 03/2010 is 1 mg/L [2]. The standard method that has been used to determine phenol levels in water according to SNI 06-6989.21-2004 is by the spectrophotometric method, in which phenol is reacted with 4-aminoantipyrine to form a brownish red complex [3]. In this method, sample preparation is needed from random measurements carried out in the laboratory. To facilitate the control of phenol levels, it is necessary to develop phenol sensors that are easily applied in the field.

Selective membranes are one of the main components in the development of chemical sensors, especially in potentiometry. Selective membranes are developed by incorporating ionophores and active ingredients in a polymer, such as polyvinyl chloride (PVC). Membranes must be a conducting material. PVC is a conducting material due to the presence of Cl^−^ which is polar and has good mechanical stability [4, 5]. Nata de coco is an example of a polar polymer because it has OH groups on their surface; hence, it can conduct electricity. Meanwhile, cellulose derived from bacteria is insoluble in water due to hydrogen bonds between adjacent hydroxyl groups [6]. In addition, bacterial cellulose has the advantage of being a membrane that has high purity, small pore size of 0.08–0.09 *μ*m, high density, low toxicity, and high mechanical stability [7, 8]. Therefore, nata de coco can be applied as a membrane in sensor manufactures. The application of nata de coco as a membrane sensor has been developed for colorimetric sensors [9, 10] and fluorosity sensors [11]; hence, it is also possible to be applied to potentiometric sensors.

**Figure 1:**
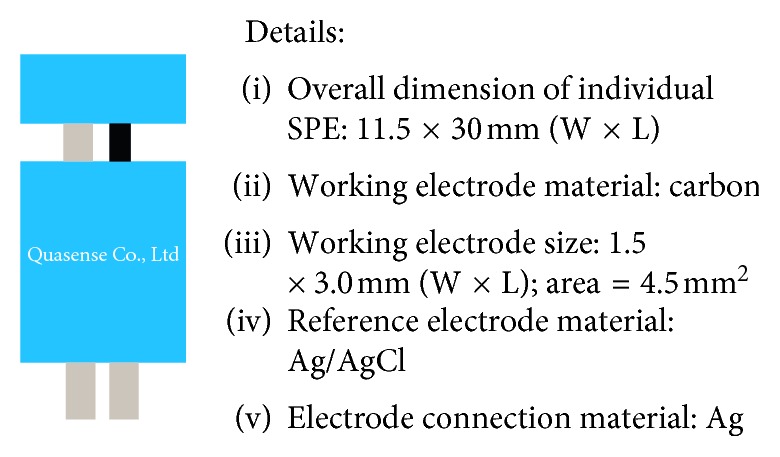
Illustrated SPE and specification used in this work. The black part on the left picture is the electrode coated with nata de coco.

Chan et al. have developed phenol sensors using PVC membranes [12]. Phenol sensors have also been developed using chitosan and cetyl trimethylammonium phenol (CTAPh) membranes as ionophores [13]. In this study, nata de coco was used as a membrane. To detect the analyte ions specifically, the same ion as the analyte must be present in the nata de coco membrane [14]. Phenoxide ion, which was deposited into the nata de coco membrane, is used as the active ion. The nata de coco selective membrane is then coated on the surface of the carbon electrode. The electrode used is a type of screen-printed electrode (SPE) consisting of a Ag/AgCl reference and an indicator electrode (Figure 1).

Signal (cell potential, E_cell_) in potentiometry is produced due to differences in concentration on the surface of the inner membrane with the outside membrane, which interacts with the analyte. The difference in concentration will cause a potential difference which is then read compared to the reference electrode [15]. Phenol is a weak acid with a pK_a_ value of 9.94 [16]; hence, phenol can be detected in the form of phenoxide ions, and their presence are influenced by pH. As an anion, the cell potential generated in the phenol sensor refers to the ESI anion, as shown in equation (1). The 0.0592 log [C_6_H_5_O^−^]_membrane_, E_asy_, and E_reff_ in equation (1) are constant; thus, the equation is simplified in equation (2):

(1) $$\begin{array}{c} {\text{E}}_{\text{cell}}=0.0592\;\log\;{\left[{{\text{C}}_{6}{\text{H}}_{5}\text{O}}^{-}\right]}_{\text{membrane}}-0.0592\;\log\;{\left[{{\text{C}}_{6}{\text{H}}_{5}\text{O}}^{-}\right]}_{\text{analyte}}{\;+\text{ E}}_{\text{asy}}-{\text{E}}_{\text{reff}} \end{array}$$

(2) $$\begin{array}{c} {\text{E}}_{\text{cell}}=\text{K}-0.0592\;\log\;{\left[{{\text{C}}_{6}{\text{H}}_{5}\text{O}}^{-}\right]}_{\text{analyte}} \end{array}$$

where K = 0.0592 log [C_6_H_5_O^−^]_membrane_–E_reff_ + E_asy_ in which E_asy_ is a potential that is unpredictable due to membrane uniformity.

**Figure 2:**
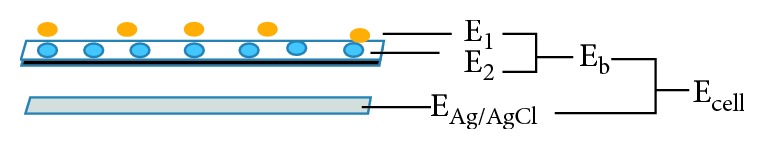
Illustrated measurement principle of phenol using the nata de coco membrane (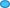 = phenol in membrane, 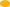 = phenol in the analyte, E_1_ = E_analyte_, E_2_ = E_membrane_, E_sel_= E_cell_, and E_b_ = the potential difference between membrane boundaries and analytes).

The difference in phenol concentration in the analyte solution with phenol concentration in the membrane is indicated from the signal or cell potential (E_cell_). The mathematical function of the relationship between concentration and signal is expressed in equation (2), which is a logarithmic equation. For monovalent ions such as phenol, sensor sensitivity is expressed by the Nernstian factor, 59.2 mV/decade. The measurement principle of the phenolic ion using the nata de coco membrane is illustrated in Figure 2 (note: E_b_ is a function of analyte concentration because the phenol concentration in the membrane is fixed. E_b_ is measured and compared to the reference electrode potential, namely, Ag/AgCl, which has a fixed amount (if using 1 M KCl solution = 222 mV).

Addition of metal oxide nanoparticles into membrane is known and been reported previously. The presence of metal oxide nanoparticles alters the properties of the membrane. For example, the hydrophobic properties and pore size of the polysulfone membrane can be modified by addition of a TiO_2_ and polyvinylpyrrolidone (PVP) mixture [17]. In our previous work, we also had successfully prepared the nata de coco membrane added with Fe_3_O_4_ on the SPCE (screen-printed carbon electrode) surface for the diazinon sensor [18]. Preparation of the Fe_3_O_4_-modified electrode for measurement of other ions or molecules in solution was also reported, such as for ascorbic acid [19], chlorite ions [20], and nitrite ions [21].

Specifically, several sensors [22] and biosensors [23, 24] have been incorporating Fe_3_O_4_ nanoparticles in their membranes. In ion selective electrodes, this nanomaterial is extensively explored as the contact material or modified materials of working electrodes due to Fe_3_O_4_ because of its electrical and hydrophobic properties [25]. Fe_3_O_4_ is believed to be able to promote rapid electron transfer between the electrode and the active site of the reaction, based on its ability to adsorb electromagnetic waves [26] and its conductivity (800 *μ*s/cm) [27].

Fe_3_O_4_ nanoparticles can also act as pseudocapacitors [28]. The capacitor is a component that can store a large electrical charge for a while [29]. Pseudocapacitors are made to increase the capacitive process in the presence of chemical reactions. This type of capacitor requires material that can chemically store the charge with a fast redox reaction. Pure Fe_3_O_4_ nanoparticles have a specific capacitance of 33 F/g, while magnetite nanoparticles coated on carbon can increase specific capacitance up to 510 F/g [30, 31]. Specific capacitance is directly proportional to the surface area, and the addition of Fe_3_O_4_ nanoparticles can increase the surface area. The existence of an increase in specific capacitance can accelerate load transduction so that it can increase the sensitivity of the sensor, which in this study was stated as the Nernstian factor.

Previously, we had develop a modified SPCE Ppy-SiO_2_ for phenol determination at a concentration of 10^3^–10^−5^ mM with a sensitivity of 7.93 *μ*A/mM [32]. In this paper, we are reporting the utilisation of nata de coco as membrane on the screen-printed electrode (SPE) for phenol degradation. The effect of the membrane thickness, pH of phenol solution, and addition of Fe_3_O_4_ nanoparticles into the membrane to the sensor performance was discussed. All preparations and measurements (except the SEM analysis) were conducted at room temperature in Chemistry Laboratory of Chemistry Department, University of Brawijaya, Indonesia.

## 2. Materials and Methods

### 2.1. Materials and Instrumentations

Chemicals were used without further purification and were all obtained from Merck, namely, phenol, NaOH, (NH_4_)_2_SO_4_, and Fe_3_O_4_ nanoparticles of 50–100 nm. Materials used in this study were nata de coco and distilled water. Laboratory tools and instrumentations used in this study were local chopper blender, magnetic stirrer, BI1703 Quasense screen-printed carbon electrode (SPCE), Sanwa CD800a potentiometer, Quasense electrode connector, Senz TI-13MO597 pH meter, Accumax Pro micropipette, Fourier-transform infrared (FT-IR) Shimadzu 8400S (4500–400 cm^−1^), and scanning electron microscopy (SEM) FEI Inspect S50 (performed at State University of Malang, Indonesia).

### 2.2. Methods

#### 2.2.1. Preparation of Phenol Sensors

Nata de coco was made by anaerobic fermentation of coconut water in the presence of *Acetobacter xylinum* for 7 days, with a composition of 1 L of coconut water, 6.7 g of sugar, 5.0 g (NH_4_)_2_SO_4_, and 100 mL of *Acetobacter xylinum* starter. A 100 g of nata de coco was cut into small pieces. Then, it was added with 50 mL of distilled water with pH 7 and blended using a blender for 5 minutes. Next, the nata de coco was filtered off using filter cloth, weighed 3 g, and added with 90 mL of distilled water with pH 7. The mixture was then ground again using a blender for 15 minutes resulting in a suspension. The suspension was then added with a 0.094 g phenol in a 10 mL of distilled water. The mixture once was again stirred at room temperature using a magnetic stirrer for 24 hours. The nata de coco membrane that has been made was finally coated onto the carbon electrode as much as 2.5, 5.0, 7.5, 10.0, 12.5, and 15.0 *μ*L (depending on the membrane thickness) and dried for 30 minutes at 50°C.

#### 2.2.2. Preparation of Phenol Sensor + Fe_3_O_4_

A 0.094 g of phenol was added with 0.1 g Fe_3_O_4_ nanoparticles and then mixed with nata de coco suspension up to 10 mL. The mixture is shaken with a shaker for 24 hours. A total of 12.5 *μ*L of the mixture was coated on the surface of the carbon electrode and dried for 30 minutes at 50°C.

#### 2.2.3. Measurement of Phenol Cell Potential

The indicator electrode was connected to the negative pole on the potentiometer, while the Ag/AgCl reference electrode was connected to the positive pole. Measurement of cell potential of the phenol solution was carried out by dripping a phenol solution on 50 *μ*L on the surface of both electrodes (indicator electrode and reference electrode). The measurement of cell potential was carried out consecutively from phenol concentrations of 10^−8^–10^−1^ M at pH 11. The cell potential was measured at room temperature for 3 minutes.

#### 2.2.4. Validation of Phenol Sensors

Four identical phenol sensors were prepared, with 100 *μ*m nata de coco membrane thickness containing phenol and Fe_3_O_4_. Each sensor was used to measure 13 phenol standard solutions at pH 11 with a concentration range of 10^−8^–10^−2^ M, and one real sample was taken from the river water. Repetition of measurements was carried out five times every five days. For validation of the river water sample, the standard addition method was used, and the calculation of sample concentration was based on the standard curve of each repetition.

#### 2.2.5. Data Analysis

A standard of deviation (S_D_) was used to validate the data. Analysis of variance was used in testing between treatments on the influence of membrane thickness with 6 treatments (*h* = 6), five replications each, total data 30 (*N*=30).

## 3. Results and Discussion

### 3.1. Preparation of Phenol Sensor

**Figure 3:**
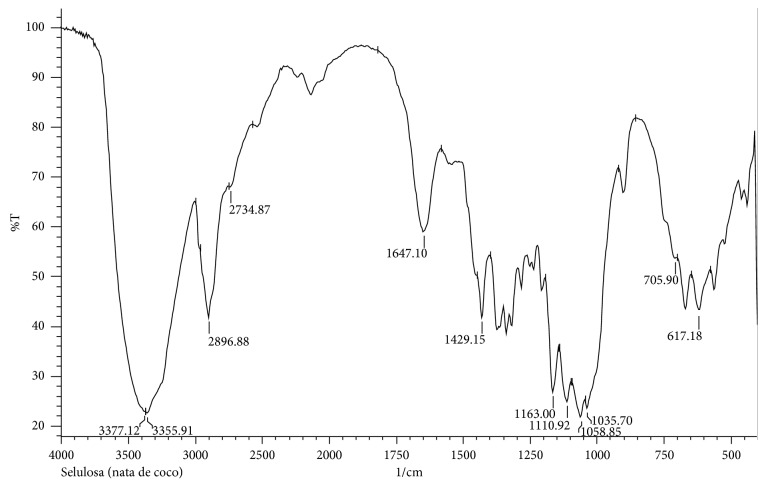
Infrared spectra of nata de coco used in this work.

One of the advantages of using nata de coco as membrane is that nata de coco has many hydroxyl groups on their surface. These functional groups, along with other functional groups, could alter the properties of the membrane, including conductivity. The hydroxyl group in nata de coco was confirmed by the broad peak around 3200–3400 cm^−1^. Nata de coco used in this work also has the alkyl (C-H) group and carbonyl (C=O) group, which was indicated by the sharp peak around 2900 cm^−1^ and 1650 cm^−1^, respectively. In detail, the infrared spectra of nata de coco are presented in Figure 3.

Phenol is an OH-substituted benzene compound, and the presence of hydroxyl (−OH) and carbonyl (C=O) groups in nata de coco allows the formation of hydrogen bonds between phenol and nata de coco. The stirring process for 24 hours is expected to result in a stronger interaction between phenol and nata de coco.

### 3.2. Effect of Nata de Coco Membrane Thickness

**Figure 4:**
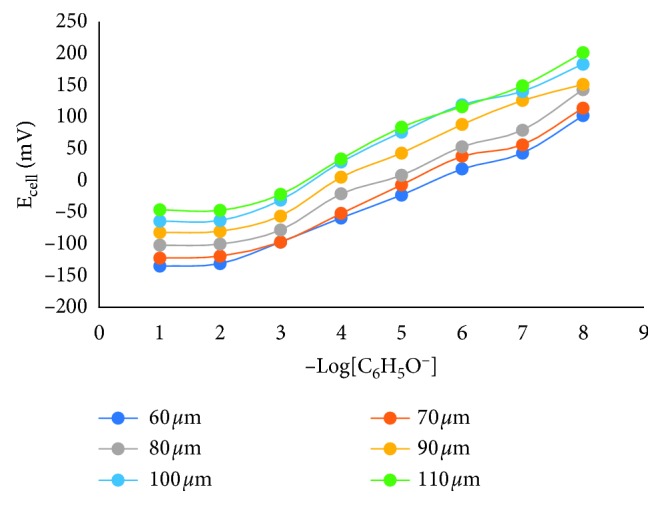
Relationship between the −log[C_6_H_5_O^−^] with the cell potential (E_cell_) at various membrane thickness.

**Figure 5:**
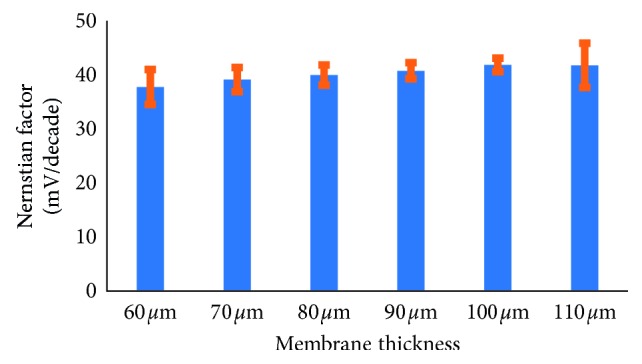
Relationship curve between membrane thickness and Nernst factor.

The nata de coco membrane thickness is studied because it will affect membrane uniformity and will affect the amount of E_asy_. In this study, the thickness of the membrane studied was 60, 70, 80, 90, 100, and 110 *μ*m. The measurements of cell potential for each membrane thickness are shown in Figure 1. As shown Figure 1, the cell potential is inversely proportional to log[C_6_H_5_O^−^], indicating that the measured sample is anion, namely, phenolic ion, and this corresponds to equation (2). All electrodes with various membrane thicknesses produce the identical pattern data, in which the signal was increasing significantly in the concentration range of 10^−2^*–*10^−8^ M. The Nernstian factor is calculated using data in Figure 4 with a concentration range of 10^−2^*–*10^−8^ M. The Nernstian factor values at various electrodes of different thicknesses are shown in Figure 5.

Sensor performance can be studied through the Nernstian factor value. For monovalent ions, the theoretical Nernstian factor is 59.2 mV/decade. Phenol sensors have a good performance if they produce a Nernstian factor close to the theoretical calculation. Figure 2 shows that the Nernstian factor increased directly and proportional to the membrane electrode thickness, except at 110 *μ*m thickness. The Nernstian factor was insignificantly increased from thickness 100 to 110 *μ*m. Thus, it was decided that the best electrodes were produced at 100 *μ*m thickness, with a Nernstian factor of 41.8 mV/decade. The increase in the Nernst factor is caused by an increase in the phenol concentration in the membrane.

As presented in Table 1, the increase in the K level (equation (2)) is straight line with the membrane thickness. This is understandable because the setting of membrane thickness was done by increasing the volume of the membrane material coated on the surface of the carbon electrode within the same area. This also shows that the membrane regularity (in this case the spread of active ions) is much better if the thickness of the membrane increases until certain limits since its thick membrane may cause irregularities. According to Fouskaki and Chaniotakis [33], a thick membrane causes the membrane surface distance with the electrode to be farther away so that the sensor response becomes slower, and this was likely to occur at the electrode with a membrane thickness of 110 *μ*m; hence, the data were less consistent, as indicated by the high SD value.

There is a slight increase in membrane electrical conductivity for each increase in membrane thickness (Table 1). This is because, in thicker membrane, the amount of water is higher, and this also results in a higher membrane electrical conductivity and capacitance [34]. In addition, the increase in membrane thickness is directly proportional to the amount of phenol in the membrane. Because phenol is a polar compound, the electrical conductivity increases with increasing phenol concentration. However, the increase in electrical conductivity has little effect on the Nernstian factor, which is a sensor performance indicator or sensor sensitivity. Thus, other effort needs to be done to increase the Nernstian factor significantly and to get closer Nernstian factor to the theoretical value.

### 3.3. Sensor Performance

**Figure 6:**
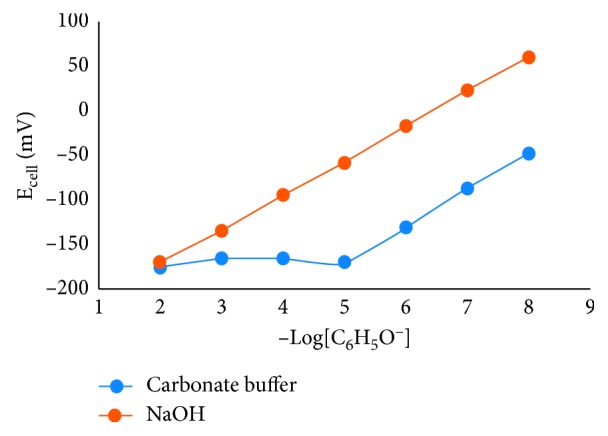
Potential cell of phenol solution at pH = 11 in carbonate and in NaOH buffers.

One of the factors that can affect the performance of the sensor is pH, considering that phenolic ions can only form perfectly (*α* = 1) at pH ≥ 10 (pKa=9.9). For this reason, the pH of the phenol solution was adjusted to pH 11 by using the 0.01 M carbonate buffer and using NaOH solution separately.  Figure 6 shows the E_cell_ difference obtained using different buffers at pH 11. As shown in Figure 6, the linear concentration was obtained in the range 10^−5^–10^−8^ M for the carbonate buffer. This result leaves uncertainty, does the presence of CO_3_^−2^ and HCO_3_^−^ ions block the interaction of phenolic ions with the membrane? The purpose of using the 0.01 M carbonate buffer is to increase the activity of the solution so that it is expected to improve the sensor performance, but the data were not inconclusive. It is possibly due to high concentration of the carbonate buffer, which is 100 times higher than the NaOH concentration in phenol solution. In addition, CO_3_^−2^ and HCO_3_^−^ ions geometrically have a larger size than Na^+^ and OH^−^ ions; thus, it is possible that the CO_3_^−2^ and HCO_3_^−^ ions inhibit the interaction of phenolic ions with the membrane.

In Figure 6, it can also be seen that the signal generated from the NaOH solution is greater than the signal generated from the 0.01 M carbonate buffer. The interaction of the electrode interface with the electrolyte solution can produce a double layer of electricity on the surface of the electrode, producing a capacitor [35–37]. In the nonstirring process, the velocity of the capacitor formation depends on the mobility of the ions naturally. Mobility of OH^−^ (20.64 × 10^−8^ m^2^s^−1^v^−1^) is higher than the mobility of CO_3_^2−^ (7.18 × 10–8 m^2^s^−1^v^−1^); hence, OH^−^ ions have a greater ability to get to the surface of the electrode [36]. This can cause the capacitance on the electrode surface to be higher in NaOH solution, than in a carbonate buffer solution, if observed at the same time. This concluded the signal produced in the NaOH solution is greater.

### 3.4. Addition of Fe_3_O_4_ Nanoparticles

**Figure 7:**
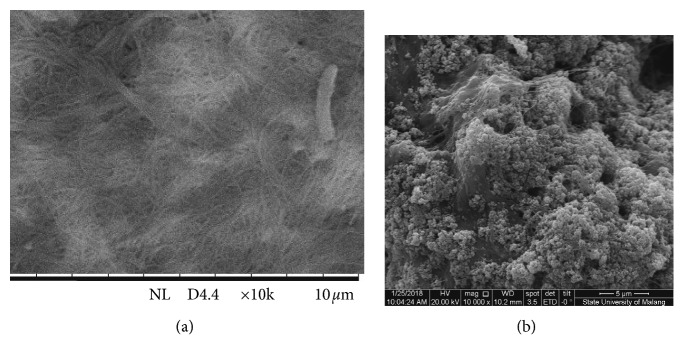
SEM images (mag: 10000x) of the sensor surface without (a) and with (b) addition of Fe_3_O_4_ nanoparticles.

Another factor that can affect sensor performance is the nature of the membrane, including selective membranes and conductive membranes. In an effort to increase membrane conductivity, Fe_3_O_4_ nanoparticles were added. The SEM images of the sensor surface with and without addition of Fe_3_O_4_ nanoparticles are presented in Figure 7. Both images show significant difference, in which nata de coco has homogeneous fibery surface, whereas due to addition of Fe_3_O_4_ nanoparticles, some of the surface area were covered with the granule shape. This also indicates that the Fe_3_O_4_ has been incorporated into the membrane. Based on SEM images, Fe_3_O_4_ makes the electrode surface to become rougher and uneven which eventually affects the sensor performance. The electrode surface profile can affect the amount of E_asy_ [15], thus affecting the value of K.

**Figure 8:**
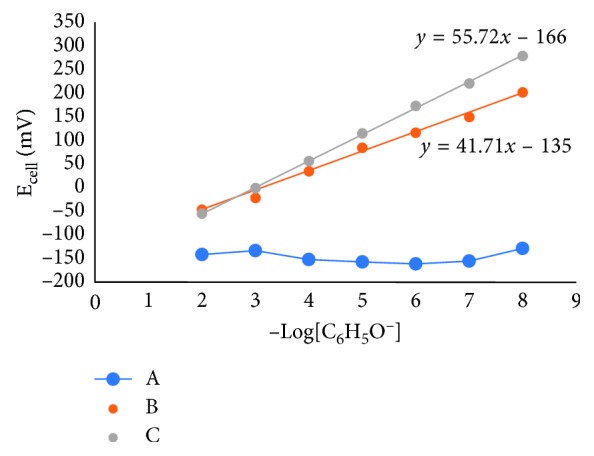
Signal data profile on changes in phenol concentration in each membrane electrodes. Electrodes with membranes made of nata de coco only (A), nata de coco + phenol (B), and nata de coco + phenol + Fe_3_O_4_ (C).

Furthermore, it is needed to investigate whether the nata de coco membrane is a selective membrane or not without the addition of phenolic ions; hence, it is necessary to also make a sensor with a membrane without phenol.  Figure 8 shows a comparison of the potential cell (signal) produced by three electrodes made of different membranes, namely, electrodes with membranes made of nata de coco only (A), made of nata de coco and phenol (B), and made of nata de coco, phenol, and Fe_3_O_4_ (C). The electrode membrane thickness of A, B, and C was set equal to 100 *μ*m.

### 3.5. Validation of Phenol Sensors

**Figure 9:**
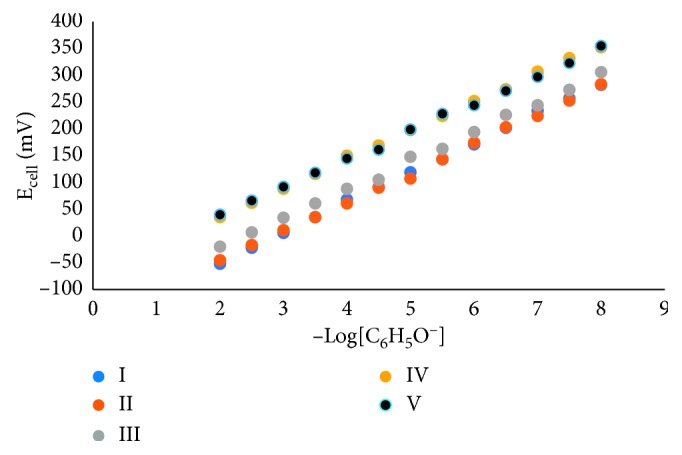
Profile of E_cell_ toward phenol concentrations at pH 11. Average signal data on first measurement (I), second repetition (II), third repetition (III), fourth repetition (IV), and fifth repetition (V).

Phenol sensor validation was carried out for 20 days using 4 identical sensors. Based on the five repetitions, the average signal of four sensors and phenol concentration are showing the same profile, as seen in Figure 9. Sensitivity, which was indicated by the value of the Nernstian factor, tends to decrease in less than 5%, except for the 5th repetition in which the decrease was up to 7%. Based on Table 2, it can be seen that phenol sensors have a good sensitivity for up to 4 times measurement, day 15 (each sensor was used to measure 64 standard phenol solutions and samples). Although the sensitivity of the sensor tends to decrease in each repetition, the accuracy of the sensor (or reproducibility of the measurement results) of the 4 sensors is reasonably very good; it can be seen from the RSD which is not more than 1%, even if the measurement results are repeated for 5 times.

The measurement accuracy can be observed from the recovery of standard samples added to river water samples. This was done so that the environmental conditions of the standard phenol electrolyte are the same as that of river water samples. As shown in Table 3, the average recovery is 93.50% for 4 *μ*M standard phenol and 93.25% for 8 *μ*M (the data are the average of the measurement obtained from 4 phenol sensors with 4 repetitions each). Based on Tables 2 and 3, it can be highlighted that phenol sensors produced from the nata de coco and Fe_3_O_4_ membrane have a good precision and accuracy.

## 4. Conclusions

Nata de coco can be used as a membrane material in a phenol sensor selective membrane by potentiometry. Membrane thickness of up to 100 *μ*m increases membrane electrical conductivity, and this corresponds to an increase in the Nernstian factor. The highest Nernstian factor was produced by electrodes with a membrane of 100 *μ*m. Setting the pH of the phenol solution to 11 using NaOH solution produces a better signal than using the carbonate buffer of 0.01 M. The addition of 0.1% Fe_3_O_4_ nanoparticles in the electrode membrane improves the sensor performance. In the concentration range of 10^−8^–10^−2^ M, the Nernstian factor increases from 41.8 ± 1.3 to 55.7 ± 0.4 mV/decade. This sensor, which was prepared from nata de coco and Fe_3_O_4_ membrane, shows good precision and accuracy.

## Figures and Tables

**Table 1:** The value of K and membrane conductivity for each membrane thickness.

No	Membrane thickness (*μ*m)	*K* (mV)	Electrical conductivity (*μ*S/cm)
1	60	−210	543
2	70	−205	545
3	80	−187	547
4	90	−164	551
5	100	−144	569
6	110	−135	561

**Table 2:** The average of Nernstian factor of 4 phenol sensors.

Days to	Nernstian factor (mV/decade)	RSD (%)	Bias (%)
1	55.71 ± 0.43	0.77	—
5	54.43 ± 0.27	0.50	2.30
10	53.75 ± 0.12	0.22	3.52
15	53.42 ± 0.48	0.90	4.12
20	51.86 ± 0.12	0.23	6.91

**Table 3:** Phenol concentration in river water sample and the recovery percentage.

Solution	Phenol concentration (*μ*M)	Recovery (%)
Sample	1.60 ± 0.04	—
Sample + 4 *μ*M phenol standard	5.34 ± 0.04	93.50 ± 0.75
Sample + 8 *μ*M phenol standard	9.06 ± 0.04	93.25 ± 0.44

## Data Availability

All data used to support the finding of this study are available from the corresponding author on reasonable request.
